# Clinical prediction tools to identify patients at highest risk of myeloma in primary care: a retrospective open cohort study

**DOI:** 10.3399/BJGP.2020.0697

**Published:** 2021-04-07

**Authors:** Constantinos Koshiaris, Ann Van den Bruel, Brian D Nicholson, Sarah Lay-Flurrie, FD Richard Hobbs, Jason L Oke

**Affiliations:** Nuffield Department of Primary Care Health Sciences, University of Oxford, Oxford, UK.; Academic Centre for Primary Care, KU Leuven, Leuven, Belgium.; Nuffield Department of Primary Care Health Sciences, University of Oxford, Oxford, UK.; Nuffield Department of Primary Care Health Sciences, University of Oxford, Oxford, UK.; Nuffield Department of Primary Care Health Sciences, University of Oxford, Oxford, UK.; Nuffield Department of Primary Care Health Sciences, University of Oxford, Oxford, UK.

**Keywords:** cancer, diagnosis, epidemiology, myeloma, primary care

## Abstract

**Background:**

Patients with myeloma experience substantial delays in their diagnosis, which can adversely affect their prognosis.

**Aim:**

To generate a clinical prediction rule to identify primary care patients who are at highest risk of myeloma.

**Design and setting:**

Retrospective open cohort study using electronic health records data from the UK’s Clinical Practice Research Datalink (CPRD) between 1 January 2000 and 1 January 2014.

**Method:**

Patients from the CPRD were included in the study if they were aged ≥40 years, had two full blood counts within a year, and had no previous diagnosis of myeloma. Cases of myeloma were identified in the following 2 years. Derivation and external validation datasets were created based on geographical region. Prediction equations were estimated using Cox proportional hazards models including patient characteristics, symptoms, and blood test results. Calibration, discrimination, and clinical utility were evaluated in the validation set.

**Results:**

Of 1 281 926 eligible patients, 737 (0.06%) were diagnosed with myeloma within 2 years. Independent predictors of myeloma included: older age; male sex; back, chest and rib pain; nosebleeds; low haemoglobin, platelets, and white cell count; and raised mean corpuscular volume, calcium, and erythrocyte sedimentation rate. A model including symptoms and full blood count had an area under the curve of 0.84 (95% CI = 0.81 to 0.87) and sensitivity of 62% (95% CI = 55% to 68%) at the highest risk decile. The corresponding statistics for a second model, which also included calcium and inflammatory markers, were an area under the curve of 0.87 (95% CI = 0.84 to 0.90) and sensitivity of 72% (95% CI = 66% to 78%).

**Conclusion:**

The implementation of these prediction rules would highlight the possibility of myeloma in patients where GPs do not suspect myeloma. Future research should focus on the prospective evaluation of further external validity and the impact on clinical practice.

## INTRODUCTION

Myeloma is the second most common haematological malignancy.^1^ In the UK the 1-year survival rate is 82.7%, 5-year survival is 52.3%, and 10-year survival is 29.1%.^2^ Myeloma mainly affects older people, with a median age at diagnosis of around 70 years.^3^^,^^4^ Delays in myeloma diagnosis are common: 50% of patients with myeloma experience an interval of >3 months between first presentation to primary care with a myeloma-related symptom and diagnosis, and they consult ≥3 times in primary care before referral to secondary care.^5^^,^^6^ Delays in diagnosis are associated with advanced-stage myeloma at diagnosis, complications, reduced disease-free survival, and poor patient-reported outcomes.^7^^–^^9^

Symptoms alone are poorly predictive of myeloma in primary care because the symptoms associated with myeloma are non-specific and common in patients without myeloma. While GPs may not think to investigate myeloma in patients with non-specific symptoms, they often order simple laboratory tests. When symptoms are combined with blood test abnormalities such as low haemoglobin, raised calcium, or raised creatinine or inflammatory markers, the risk of myeloma increases^10^^,^^11^ and the National Institute for Health and Care Excellence recommends definitive cancer investigation.^12^ Furthermore, certain blood tests such as low haemoglobin can be observed up to 2 years before a myeloma diagnosis, providing a potential window of opportunity for earlier diagnosis.^10^^,^^11^

Clinical prediction tools for myeloma are quite limited. Currently, the only one that exists in primary care is based on a Clinical Practice Research Datalink (CPRD) study, in which the authors report the positive predictive values of single/paired symptoms and investigations.^10^ Another study generated a prediction rule that could be useful for myeloma, but it was developed in hospitalised patients and the outcome was not confirmed diagnosis of myeloma but abnormal serum/urine protein electrophoresis.^13^ The aim of this study, therefore, was to develop novel prediction rules that combine symptoms and blood tests to identify people attending primary care who are at increased risk of myeloma, with a focus on the most commonly requested blood test group in primary care, the full blood count (FBC).

## METHOD

A retrospective open cohort study was conducted using electronic health records data from the CPRD, a representative primary care database that includes 11.3 million patients from 674 practices in the UK.^14^ People were included in the study if they were aged ≥40 years, had been registered with their practice for at least 1 year, and had at least two FBC tests recorded within 1 year (at least one FBC component recorded: haemoglobin, mean corpuscular volume [MCV], platelets, or white cell count) between 1 January 2000 and 1 January 2014, and for whom a minimum-follow up of 2 years was available. The start of the follow-up was from the date of the second FBC test (index date).

**Table:** How this fits in

Multiple myeloma is a haematological cancer in which 50% of patients experience symptoms for at least 3 months before diagnosis and have multiple consultations in primary care before referral to secondary care. Symptoms on their own are not predictive enough to suggest referral and they have to be combined with abnormalities in blood tests. The authors of the present study developed two clinical prediction rules that combine patient characteristics, symptoms, and common blood tests to identify patients at high risk of having undiagnosed myeloma. The study found that the prediction rules were shown to have good discrimination, and have the potential to reduce the delays observed in the diagnosis of myeloma.

Patients who had been diagnosed with myeloma or monoclonal gammopathy of undetermined significance (MGUS) before the index date were excluded from the study. Patients with MGUS were excluded as they are usually monitored quite closely for progression to symptomatic myeloma because their risk of progressing is approximately 1% per year, which is markedly higher than the baseline risk in the population.^15^ End of follow-up was the earliest of 2 years’ follow-up or myeloma diagnosis.

### Predictors and outcome

Possible predictors for myeloma were identified from the literature including demographics (age, sex, and body mass index [BMI]), symptoms (back, chest, bone, rib, and joint pain, shortness of breath, recurrent chest infections, fatigue, nosebleeds, bruising, fracture, weight loss, and nausea), and blood test results (FBC components, inflammatory markers: erythrocyte sedimentation rate [ESR], C-reactive protein (CRP), and plasma viscosity (PV), calcium, and creatinine). Myeloma was defined as a new diagnosis of myeloma within 2 years of the index date using a code in the electronic health records (Read code).

### Sample size

Twenty or more events per variable is adequate to eliminate bias in Cox models when there are many low prevalence predictor variables.^16^ With 25 candidate predictor variables and an event rate of 20 events per variable, it was estimated that 500 events were necessary for the derivation dataset. Validation datasets should ideally be ≥200.^17^

### Statistical analysis

The dataset was split into derivation and validation sets based on English geographical region. Two-thirds were assigned to the derivation dataset and one-third to the validation dataset.^14^ Descriptive statistics were used to summarise the baseline characteristics, predictor variables, and outcomes. Diagnostic accuracy measures (sensitivity, specificity, positive and negative likelihood ratios, and positive and negative predictive values) were calculated for individual and combined symptoms, and for blood test results.

Multiple imputation was used to address missing data. Ten imputations were created for the derivation and external validation datasets separately. Imputation models contained all the predictors, the binary indicator for the outcome, and the cumulative baseline hazard estimated by the Nelson–Aalen estimator.^18^ Continuous variables were centred and rescaled to help with the convergence of the models. Fractional polynomials were used to identify the optimal functional form of continuous variables: BMI, age, and blood test results.^19^ Univariable analysis was used a priori to select the inflammatory marker with the highest hazard ratio for inclusion in the multivariable analysis, as inflammatory marker results are highly correlated. In sensitivity analyses blood test results were classified as normal/abnormal (instead of modelling continuously) depending on the reference range provided by the local laboratory. Normocytic anaemia was defined as low haemoglobin and normal MCV. Macrocytic anaemia was defined as high MCV and low haemoglobin.

### Model derivation

Starting with the following variables: demographics (age, sex, and BMI), symptoms (back, chest, bone, rib, and joint pain, shortness of breath, recurrent chest infections, fatigue, nosebleeds, bruising, fracture, weight loss, and nausea), and blood test results (FBC components, inflammatory markers — ESR, CRP, and PV — calcium, and creatinine), the mfpmi command in Stata (version 14) was used to select variables for inclusion in Cox proportional hazards models using backwards elimination with a 5% inclusion.^20^ For the derivation, multivariable Cox proportional hazards models were fitted as follows:

- FBC model: demographics, symptoms, and FBC components;
- FBC change model: demographics, symptoms, and the absolute change between the index FBC test and previous FBC test; and
- all-test model: demographics, symptoms, and all tests currently used for myeloma diagnosis (FBC components, calcium, creatinine, and inflammatory markers).

### External validation

The baseline survival function was calculated at 2 years using Kaplan–Meier estimates and combined with the regression coefficients to derive the final equations. These equations were used to predict the probability of myeloma in the external validation dataset. Model performance was examined in terms of calibration, discrimination, and clinical usefulness. Calibration was assessed by the use of calibration plots, and discrimination using the *R*^2^ statistic, the D statistic, and the area under the curve (AUC).^21^^,^^22^ Decision curve analysis was used to compare the clinical utility of the models.^23^ Diagnostic accuracy measures were then estimated for the various cut-offs of myeloma probability.

## RESULTS

A total of 1 281 926 patients were included, with a mean age of 63.7 years (SD 13.8), of whom 41.1% were male. The derivation and validation sets were comparable in terms of age, sex, risk factors, symptoms, and blood tests (see Supplementary Table S1 for details). A total of 737 incident myeloma cases (0.06%) were diagnosed within 2 years: 495 (0.06%) in the derivation set, and 242 (0.05%) in the validation set.

### Symptoms and blood tests

The most common symptoms recorded for myeloma patients were back pain (19.0% versus 9.4% in non-myeloma) and chest pain (11.3% versus 6.4% in non-myeloma) (Table 1). Anaemia (irrespective of type) was the most common abnormality observed in the FBC (58.1% compared with 15.3% in nonmyeloma) and high MCV with prevalence of 20.0% compared with 6.9% in non-myeloma. Of the inflammatory markers, ESR was most frequently abnormal in patients with myeloma (80.1%). Inflammatory markers, calcium, and creatinine had the highest fractions of missing data. In the derivation dataset, 46.4% of patients with myeloma had anaemia at both tests, with the average time between the two abnormal tests being 2 months. The median time to myeloma diagnosis from the index test was 5.6 months (interquartile range = 1.6 to 15.7) (data not shown).

**Table 1.** Descriptive statistics for derivation dataset

**Variable**	**Missing data (*n* = 835 404), *n* (%)^a^**	**Non-myeloma (*n*= 834 909), *n* (%)^a^**	**Myeloma (*n*= 495), *n* (%)^a^**
**Demographics**			
Female	0 (0.0)	492 039 (58.9)	227 (45.9)
Mean age, years (SD)	0 (0.0)	63.7 (13.8)	70.9 (10.5)
Mean BMI (SD)	541 782 (64.9)	28.5 (6.2)	26.5 (4.6)

**Symptoms**			
Back pain	0 (0.0)	78 291 (9.4)	94 (19.0)
Chest pain	0 (0.0)	53 256 (6.4)	56 (11.3)
Bone pain	0 (0.0)	12 933 (1.5)	12 (2.4)
Rib pain	0 (0.0)	3809 (0.5)	8 (1.6)
Joint pain	0 (0.0)	35 348 (4.2)	19 (3.8)
Shortness of breath	0 (0.0)	66 047 (7.9)	53 (10.7)
Chest infections	0 (0.0)	54 198 (6.5)	40 (8.1)
Fatigue	0 (0.0)	66 903 (8.0)	34 (6.9)
Nosebleeds	0 (0.0)	7022 (0.8)	14 (2.8)
Bruising	0 (0.0)	8682 (1.0)	5 (1.0)
Fracture	0 (0.0)	13 361 (1.6)	12 (2.4)
Weight loss	0 (0.0)	10 985 (1.3)	12 (2.4)
Nausea	0 (0.0)	25 126 (3.0)	21 (4.2)

**Blood tests**			
2nd FBC test (index)			
Mean haemoglobin (SD)	50 292 (6.0)	13.5 (1.6)	12.0 (1.9)
Mean white cell count (SD)	62 843 (7.5)	7.3 (5.2)	6.5 (3.6)
Mean platelets (SD)	67 222 (8.0)	265.4 (80.2)	247.4 (80.6)
Mean MCV (SD)	72 537 (8.7)	90.6 (5.9)	93.5 (6.1)

**Difference in FBC parameters (2nd – 1st)**			
Mean haemoglobin diff (SD)	87 941 (10.5)	0.01 (1.0)	−0.25 (1.1)
Mean white cell count diff (SD)	101 091 (12.1)	−0.04 (5.3)	0.13 (2.3)
Mean platelets diff (SD)	107 485 (12.9)	−1.8 (54.9)	−2.9 (61.1)
Mean MCV diff (SD)	114 841 (13.7)	0.19 (3.1)	−0.06 (2.4)

**Other tests**			
Mean calcium (SD)	634 969 (76.0)	2.3 (0.12)	2.4 (0.18)
Mean creatinine (SD)	453 831 (54.3)	89.3 (30.2)	99.9 (44.9)
Mean ESR (SD)	621 155 (74.4)	18.6 (19.5)	55.3 (41.7)
CRP, median (IQR)	679 841 (81.4)	5 (2 to 10)	5 (2.5 to 17)
Mean PV (SD)	798 298 (95.6)	1.71 (0.16)	1.96 (0.73)

**Blood tests (normal/abnormal)^b^**			
**FBC (index test)**			
Anaemia	50 292 (6.0)	120 247/784 649 (15.3)	269/463 (58.1)
Leukopenia	62 843 (7.5)	22 424/772 119 (2.9)	59/442 (13.3)
Low platelets	67 222 (8.0)	25 165/767 738 (3.3)	44/444 (9.9)
High MCV	72 537 (8.7)	52 385/762 443 (6.9)	85/424 (20.0)

**Other tests^b^**			
Abnormal calcium	634 969 (76.0)	4056/200 263 (2.0)	14/172 (8.1)
High creatinine	453 831 (54.3)	56 106/381 341 (14.7)	63/232 (27.2)
High ESR	621 155 (74.4)	89 735/214 088 (41.9)	129/161 (80.1)
High CRP	679 841 (81.4)	51 000/155 452 (32.8)	47/111 (42.3)
High PV	798 298 (95.6)	14 080/37 084 (38.0)	15/22 (68.2)

a Unless otherwise stated.

b Percentages reported for patients with complete data. Denominator displayed to indicate where missing data applies. BMI = body mass index. CRP = C-reactive protein. ESR = erythrocyte sedimentation rate. FBC = full blood count. IQR = interquartile range. MCV = mean corpuscular volume. PV = plasma viscosity. SD = standard deviation.

### Prediction model derivation and validation

**Figure 1.**
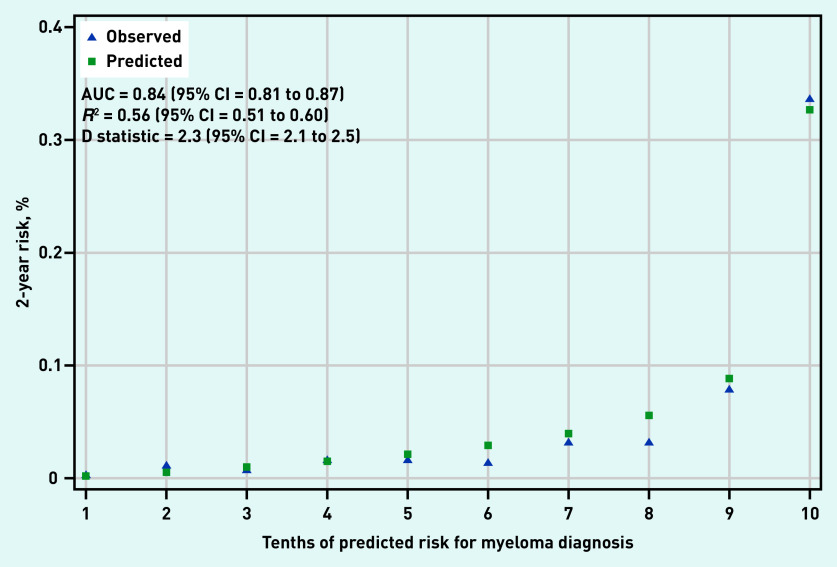
***Calibration and discrimination of full blood count model.*** ***AUC = area under curve.***

**Figure 2.**
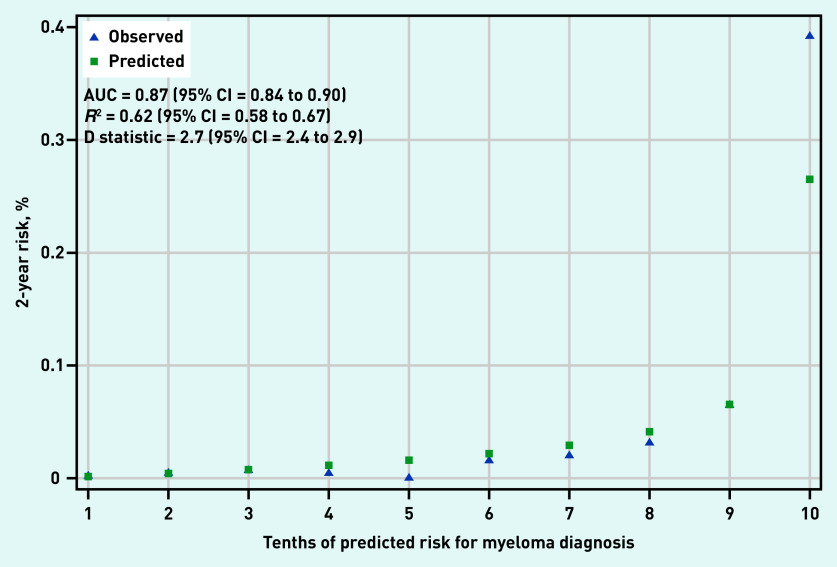
***Calibration and discrimination of all-test model.*** ***AUC = area under curve.***

Back pain, chest pain, rib pain, nosebleeds, and all FBC parameters were selected for inclusion in both the FBC and all-test model (Table 2). The FBC-change model was dropped because FBC change parameters were not selected for inclusion in the final model. In the validation dataset, the FBC model had an AUC of 0.84 (95% confidence interval [CI] = 0.81 to 0.87) (Figure 1) and the all-test model had an AUC of 0.87 (95% CI = 0.84 to 0.90) (Figure 2). The D statistic values were 2.3 (95% CI = 2.1 to 2.5) and 2.7 (95% CI = 2.4 to 2.9) for the FBC model and all-test model, respectively. Similarly, *R*^2^ values were 0.56 (95% CI = 0.51 to 0.60) and 0.62 (95% CI = 0.58 to 0.67) for the FBC model and all-test model, respectively. For reference, D statistic values of 0 correspond to a model with an AUC of 0.5, while values ≥3 correspond to models with an AUC >0.9.^24^ Calibration plots showed good agreement between predicted and observed risk in both the FBC and all-test model (Figures 1 and 2). However, the all-test model under-predicted myeloma risk in the highest decile (Figure 2).

**Table 2.** Adjusted hazard ratios (95% CI) for the final models for myeloma

**Variable**	**FBC model,^a^**	**All-test model,^b^**
	**HR (95% CI)**	**HR (95% CI)**
**Demographics**		
Female	0.45 (0.37 to 0.54)	0.48 (0.39 to 0.58)
Age^c^	FP (0.5, 0.5)	FP (0.5, 0.5)

**Symptoms**		
Back pain	2.37 (1.89 to 2.98)	2.46 (1.96 to 3.10)
Chest pain	1.76 (1.33 to 2.33)	1.85 (1.39 to 2.45)
Rib pain	2.94 (1.46 to 5.99)	2.81 (1.38 to 5.72)
Nosebleeds	2.26 (1.32 to 3.85)	2.11 (1.23 to 3.61)

**FBC**		
Haemoglobin^c^	FP (3, 3)	FP (3, 3)
White cell count^c^	FP (−2, −2)	FP (−2, −2)
Platelets^c^	FP (−1, −0.5)	FP (−0.5, 0)
MCV^c^	FP (3, 3)	FP (3, 3)

**Other tests**		
ESR	NA	1.03 (1.03 to 10.33)
Calcium^c^	NA	FP (−1)

a FBC model contains a single FBC.

b All-test model contains a single FBC, plus ESR and calcium.

c *Numerals in parenthesis represent the transformations used.* *CI = confidence interval. ESR = erythrocyte sedimentation rate.*

FBC = full blood count. FP = fractional polynomials. HR = hazard ratio. MCV = mean corpuscular volume. NA = not applicable.

### Diagnostic accuracy and comparison of different diagnostic approaches

Table 3 presents diagnostic accuracy measures for symptoms, blood tests, their combinations, and a range of predicted myeloma probability. Anaemia (irrespective of type) had a sensitivity of 56% (95% CI = 49% to 63%), a specificity of 83% (95% CI = 83% to 84%), and a positive predictive value of 0.18% (95% CI = 0.15% to 0.21%). The FBC and the all-test clinical prediction rules, using the 90th percentile of the predicted probability, resulted in sensitivities of 62% (95% CI = 55% to 68%) and 72% (95% CI = 66% to 78%), respectively, specificities of 90% (95% CI = 90% to 90%) for both models, and positive predictive values of 0.34% (95% CI = 0.29% to 0.40%) and 0.40% (95% CI = 0.34% to 0.47%), respectively. Decision curve analysis showed that, independently of which threshold is used for the models, decisions made using the prediction models result in fewer false positives and more true positives when compared with single tests or symptoms (see Supplementary Figure S1 for details).

**Table 3.** Comparison of different diagnostic approaches in the validation cohort (after performing imputation)^a^

**Variable**	**Pr, %^b^**	**Sensitivity, % (95% CI)**	**Specificity, % (95% CI)**	**LR+ (95% CI)**	**LR– (95% CI)**	**PPV, % (95% CI)**	**NPV, % (95% CI)**
**Symptoms**							
Back pain	NA	21.5 (16.5 to 27.2)	91.2 (91.1 to 91.3)	2.4 (1.9 to 3.1)	0.86 (0.81 to 0.92)	0.13 (0.10 to 0.17)	99.95 (99.94 to 99.96)
Rib pain	NA	1.2 (0.3 to 3.6)	99.5 (99.5 to 99.5)	2.5 (0.8 to 7.7)	0.99 (0.99 to 1.0)	0.14 (0.01 to 0.29)	99.94 (99.94 to 99.95)
Chest pain	NA	9.1 (5.8 to 13.4)	93.6 (93.6 to 93.7)	1.4 (0.9 to 2.1)	0.97 (0.93 to 1.0)	0.08 (0.05 to 0.11)	99.95 (99.94 to 99.95)
Nosebleeds	NA	1.2 (0.3 to 3.6)	99.2 (99.2 to 99.2)	1.6 (0.5 to 4.9)	0.99 (0.98 to 1.0)	0.09 (0.01 to 0.18)	99.94 (99.94 to 99.95)

**FBC (index test)**							
Anaemia (any type)	NA	55.6 (48.6 to 62.5)	83.6 (83.5 to 83.7)	3.4 (3.2 to 3.6)	0.53 (0.39 to 0.67)	0.18 (0.15 to 0.21)	99.97 (99.96 to 99.98)
Normocytic anaemia	NA	43.5 (37.0 to 50.0)	85.1 (84.9 to 85.2)	2.9 (2.7 to 3.2)	0.66 (0.54 to 0.77)	0.15 (0.13 to 0.19)	99.96 (99.95 to 99.97)
Macrocytic anaemia	NA	11.9 (8.1 to 15.8)	98.5 (98.4 to 98.6)	8.1 (6.3 to 9.8)	0.89 (0.84 to 0.95)	0.43 (0.26 to 0.61)	99.95 (99.94 to 99.96)
Low platelets	NA	8.3 (5.5 to 11.1)	96.5 (96.4 to 96.6)	2.4 (1.9 to 2.9)	0.95 (0.91 to 0.98)	0.13 (0.07 to 0.18)	99.95 (99.94 to 99.96)
Low WCC	NA	7.0 (4.5 to 9.6)	96.9 (96.9 to 97.0)	2.3 (1.7 to 2.9)	0.96 (0.93 to 0.99)	0.13 (0.06 to 0.19)	99.94 (99.94 to 99.95)
High MCV	NA	22.9 (17.5 to 28.4)	91.6 (91.5 to 91.7)	2.8 (2.3 to 3.2)	0.84 (0.76 to 0.92)	0.15 (0.10 to 0.19)	99.95 (99.94 to 99.96)

**Other tests**							
Hypercalcemia	NA	5.7 (1.9 to 9.6)	98.3 (98.2 to 98.4)	3.4 (1.3 to 5.5)	0.96 (0.91 to 1.0)	0.19 (0.04 to 0.33)	99.95 (99.94 to 99.96)
High ESR	NA	80.3 (70.4 to 90.3)	54.8 (54.5 to 55.1)	1.7 (1.6 to 1.9)	0.35 (0.08 to 0.63)	0.10 (0.08 to 0.11)	99.98 (99.97 to 99.99)
High ESR or anaemia	NA	90.5 (81.4 to 99.5)	50.0 (49.8 to 50.3)	1.8 (1.7 to 1.9)	0.19 (0.13 to 0.28)	0.10 (0.08 to 0.12)	99.99 (99.98 to 99.99)
High ESR and anaemia	NA	45.5 (38.9 to 51.9)	88.4 (88.2 to 88.5)	3.9 (3.6 to 4.2)	0.62 (0.50 to 0.74)	0.20 (0.17 to 0.25)	99.96 (99.96 to 99.97)

**FBC model**							
77th percentile^c^	0.06	78.9 (73.2 to 83.9)	77.2 (77.1 to 77.3)	3.5 (3.2 to 3.7)	0.27 (0.21 to 0.35)	0.19 (0.16 to 0.22)	99.98 (99.97 to 99.98)
90th percentile	0.12	61.6 (55.1 to 67.7)	90.2 (90.1 to 90.3)	6.3 (5.7 to 6.9)	0.43 (0.36 to 0.50)	0.34 (0.29 to 0.40)	99.98 (99.97 to 99.98)
95th percentile	0.20	41.3 (35.1 to 47.8)	95.1 (95.0 to 95.2)	8.4 (7.3 to 9.8)	0.62 (0.55 to 0.69)	0.46 (0.37 to 0.55)	99.96 (99.96 to 99.97)
99th percentile	0.60	18.2 (13.5 to 23.6)	99.1 (99.1 to 99.1)	19.9 (15.2 to 26.1)	0.83 (0.78 to 0.88)	1.10 (0.80 to 1.40)	99.95 (99.95 to 99.96)
99.5th percentile	0.90	12.8 (8.9 to 17.7)	99.5 (99.5 to 99.6)	27.6 (19.8 to 38.4)	0.88 (0.84 to 0.92)	1.50 (1.00 to 2.10)	99.95 (99.95 to 99.96)
99.9th percentile	2.20	4.1 (2.0 to 7.5)	99.9 (99.9 to 99.9)	42.4 (23.5 to 82.9)	0.96 (0.94 to 0.99)	2.30 (1.10 to 4.10)	99.95 (99.94 to 99.95)

**All-test model**							
84th percentile^c^	0.06	82.6 (77.3 to 87.2)	83.9 (83.8 to 84.0)	5.1 (4.8 to 5.4)	0.21 (0.16 to 0.27)	0.28 (0.24 to 0.32)	99.98 (99.98 to 99.99)
90th percentile	0.09	71.9 (65.8 to 77.5)	90.3 (90.2 to 90.4)	7.4 (6.9 to 8.0)	0.31 (0.25 to 0.38)	0.40 (0.34 to 0.47)	99.98 (99.97 to 99.98)
95th percentile	0.15	62.4 (56.8 to 68.5)	95.1 (95.0 to 95.1)	12.9 (11.7 to 14.2)	0.40 (0.34 to 0.47)	0.70 (0.59 to 0.81)	99.98 (99.97 to 99.98)
99th percentile	0.45	34.3 (28.3 to 40.6)	99.0 (99.0to 99.1)	35.1 (29.4 to 41.9)	0.66 (0.61 to 0.73)	1.9 (1.5 to 2.3)	99.96 (99.96 to 99.97)
99.5th percentile	0.70	24.0 (18.7 to 29.9)	99.5 (99.5 to 99.5)	48.9 (38.9 to 61.4)	0.76 (0.71 to 0.82)	2.6 (2.0 to 3.3)	99.96 (99.95 to 99.96)
99.9th percentile	1.90	7.8 (4.8 to 12.0)	99.9 (99.9 to 99.9)	80.5 (51.8 to 125.0)	0.92 (0.89 to 0.96)	4.2 (2.5 to 6.4)	99.95 (99.94 to 99.95)

a Results presented are based on multiple imputation as described in the methods section.

b Pr = corresponding probability (%) of the selected risk score percentile.

c These percentile values were selected to match the background prevalence in the whole cohort. ESR = erythrocyte sedimentation rate. FBC = full blood count. LR– = negative likelihood ratio. LR+ = positive likelihood ratio. MCV = mean corpuscular volume. NA = not applicable. NPV = negative predictive value. PPV = positive predictive value. WCC = white cell count.

Table 4 shows the performance of different diagnostic approaches assuming a population of 100 000 tested patients. The FBC model at the 90th percentile threshold of risk (0.12%) would result in 270 false alarms to one myeloma case diagnosed and in one missed myeloma case to 3910 true negatives. Comparatively, investigating based on anaemia would result in 500 false alarms per myeloma case and in one missed myeloma case to 3190 true negatives. Overall, the number of false positives will be lower using the rule at almost all thresholds compared with all other approaches that use single symptoms or tests. High calcium, low platelets, and low white cell count had very high specificity values (>95%) but low sensitivity values (<10%) (Table 3), which results in few false positives but many missed cases of myeloma.

**Table 4.** Performance of the different diagnostic approaches in a population of 100 000 tested individuals based on the validation cohort measures

**Variable**	**Per 100 000 patients (60 myeloma cases), *n***				**Ratio of false alarms to cancers diagnosed**	**Ratio of true negatives to cancers missed**

	**Cancers diagnosed**	**False alarms**	**Cancers missed**	**Correctly spared investigations**		
**Symptoms**						
Back pain	13	8995	47	90 945	692 to 1	1935 to 1
Chest pain	5	5996	55	93 944	1199 to 1	1708 to 1
Rib pain	1	500	59	99 440	500 to 1	1685 to 1
Nosebleeds	1	800	59	99 140	800 to 1	1680 to 1

**FBC (index test)**						
Anaemia (any type)	34	16 990	26	82 950	500 to 1	3190 to 1
Low platelets	5	2998	55	96 942	600 to 1	1763 to 1
Low WCC	4	2999	56	96 941	750 to 1	1731 to 1
High MCV	14	7995	46	91 945	571 to 1	1998 to 1

**Other tests**						
Hypercalcemia^a^	4	1999	56	97 941	500 to 1	1748 to 1
High ESR^a^	48	44 973	12	54 967	936 to 1	4581 to 1
High ESR or anaemia^a^	54	49 970	6	49 970	925 to 1	8328 to 1
High ESR and anaemia^a^	27	11 993	33	87 947	444 to 1	2665 to 1

**FBC model**						
Prevalence	48	22 986	12	76 954	479 to 1	6412 to 1
90th percentile	37	9994	23	89 946	270 to 1	3910 to 1
95th percentile	25	4997	35	94 943	200 to 1	2712 to 1
99th percentile	11	999	49	98 941	91 to 1	2019 to 1

**All-test model^a^**						
Prevalence	50	16 090	10	83 850	322 to 1	8385 to 1
90th percentile	43	9994	17	89 946	232 to 1	5290 to 1
95th percentile	37	4997	23	94 943	135 to 1	4127 to 1
99th percentile	20	999	40	98 941	25 to 1	2473 to 1

a Corresponds to the performance measures if ESR and calcium were to be ordered for all patients in the sample. ESR = erythrocyte sedimentation rate. FBC = full blood count.

MCV = mean corpuscular volume. WCC = white cell count.

## DISCUSSION

### Summary

In this study, two clinical prediction models were generated to predict myeloma risk and raise the suspicion of myeloma in patients who are tested with FBC and for whom the GP does not necessarily suspect myeloma. The FBC model includes age, sex, back, chest, and rib pain, nosebleeds, and components of the FBC. The all-test model also includes ESR and calcium. The two models were validated in patients attending primary care. Both models discriminated well between people with and without myeloma, but the FBC model was better calibrated than the all-test model. Choosing to investigate people classified in the top decile of predicted myeloma risk (0.12%) would lead to fewer false alarms for each case of myeloma investigated compared with selecting people based on symptoms or blood test abnormalities alone.

### Strengths and limitations

These prediction models are immediately relevant to myeloma diagnosis in primary care as they were developed using data from routinely collected primary care records. A split-sample approach, based on geographical region, allowed for meaningful validation and increases the likelihood of model reproducibility in other datasets from primary care. By assessing discrimination, calibration, the performance of the models at different thresholds, and comparing them with single-test approaches using diagnostic accuracy measures and decision curve analysis, this study has demonstrated the benefits of using a prediction modelling approach over decision rules based on symptoms or tests alone.

This study has several limitations. The population was selected based on two FBCs in order to assess whether change between two FBC components was predictive of myeloma. The change variables were not significant, which could be attributed to the fact that in many cases blood test abnormalities were being detected at both tests, that is, patients were presenting with anaemia on multiple occasions. This is likely because abnormalities in the first test are correlated with the likelihood of having a second test. An English study found that 23.5% of primary care patients aged >65 years had two FBCs over a period of 2 years, suggesting that patients in the current study are a selected population and more likely to represent a sicker population.^25^ It has also been shown that patients who have blood tests are more likely to have cancer.^26^ Following abnormalities in the initial FBC, 46.4% of patients with myeloma could have been picked up if investigated at that timepoint. The prediction rules can be applied to this population in order to identify which patients should be further investigated for myeloma at the time of the first test, thus shortening the diagnostic process. The prediction rules developed in this study should be validated further in different populations, such as patients receiving one FBC rather than two, and potentially in other countries in order to confirm their generalisability.

As coding in routine health records is done for clinical purposes, it is influenced by the variability in history taking and recording behaviour between primary care clinicians. It is likely that patients do not report all of their symptoms and also that GPs may only record the symptom(s) they consider the most relevant, especially for myeloma symptoms, which are often quite vague and low risk. To what extent this happens in practice and how it affects the accuracy of the prediction models is unclear.

The all-test model had a large proportion of missing data because calcium and ESR recordings were only available for a small number of patients. This meant that only 8% of the whole sample would be included in a complete case analysis. Multiple imputation was used to avoid limiting the analysis but the reason for missingness may have not been accounted for in the imputation model. Furthermore, the number of imputations might not have been sufficient given the large fraction of missing data, but the large sample size meant that additional imputations would have been computationally prohibitive.

Finally, there was no linkage with Hospital Episode Statistics data or cancer registry data, thus there is a lack of formal outcome ascertainment. The accuracy, quality, and completeness of CPRD data has been validated previously.^27^

### Comparison with existing literature

To the authors’ knowledge, this is the largest retrospective open cohort study to develop prediction rules for myeloma in primary care. The prediction models in this study perform similarly to established prediction rules for cancer.^28^^,^^29^ The findings in the current study regarding the utility of a normal ESR and normal haemoglobin for ruling out myeloma confirm those of previous primary care studies.^10^^,^^11^

### Implications for research and practice

This study presents the diagnostic accuracy of multiple thresholds of predicted myeloma risk to illustrate rule-in and rule-out approaches by maximising specificity or sensitivity. The authors recommend selecting a threshold with a specificity >90%, such as the 90th percentile of the FBC model, leading to more true positives and fewer false positives compared with other approaches, such as acting on anaemia alone. More specifically, at the 90th percentile threshold of risk, the rule would diagnose an extra 18% of patients compared with normocytic anaemia and an extra 6% of patients compared with anaemia of any type (normocytic, microcytic, or macrocytic), with fewer false positives (estimated based on data in Table 3). While other blood tests such as calcium have higher specificity, resulting in fewer false positives, their sensitivity is much lower, meaning that many cancers would be missed. Previous studies have shown that hypercalcaemia develops later in disease progression; thus, while predictive of myeloma, it is less useful for detecting myeloma early.^11^ The median time to myeloma diagnosis from the index test (second FBC) is 5.6 months (interquartile range = 1.6 to 15.7), suggesting that the prediction rules have the potential to reduce diagnostic delays by a substantial amount.

The prediction rules devised in this study are able to raise the suspicion of myeloma in patients who are regularly tested with FBC either for monitoring purposes or as part of a diagnostic process. Patients who are flagged as being at high risk of having myeloma can be tested with serum and urine protein electrophoresis in primary care, and abnormalities in these tests should result in a haematology referral. Nonetheless, myeloma can be missed even with the use of a prediction rule, subject to the decision threshold that is used and the corresponding sensitivity, so in these patients other follow-up tests could potentially be used, such as ESR or PV, if it is indicated by the clinical presentation of the patient.

As these prediction rules are complex scoring systems, they require software. This could be a web-based calculator or could be integrated within the electronic health records of general practices to trigger alerts to GPs about patients with a high predicted risk of myeloma, or to the local laboratory to automatically process or request a myeloma screen. Electronic trigger interventions have been shown to reduce diagnostic delays in colorectal and prostate cancer.^30^ Future research should explore the feasibility of such a tool, identify and explore the different barriers that might prevent its implementation, and establish its acceptability. Impact studies are recommended to explore the effect of the prediction rule on the diagnostic pathway and on important outcomes such as stage at diagnosis and survival.
